# Extensor Carpi Radialis Longus and Brevis Rupture in a Boxer

**Published:** 2014-10-21

**Authors:** T. Mundell, N. Miladore, T. Ruiter

**Affiliations:** ^a^Oakland University William Beaumont School of Medicine, Rochester, MI; ^b^Department of Orthopedics, Western Michigan University Homer Stryker MD School of Medicine, Kalamazoo, MI

**Keywords:** extensor carpi radialis longus, extensor carpi radialis brevis, avulsion, rupture, suture anchor

## DESCRIPTION

A 34-year-old man experienced rupture of his extensor carpi radialis longus (ECRL) and extensor carpi radialis brevis (ECRB) tendons after striking a heavy boxing bag. Magnetic resonance imaging demonstrated an empty second extensor compartment and avulsion of the extensor tendons from their insertion sites. Both tendons were explored and reinserted using mini-suture anchors.

## QUESTIONS

1. **What is the incidence of ECRL/ECRB avulsion injuries?**
2. **What risk factors exist for tendon avulsion injuries?**
3. **What is the pullout strength of suture anchors?**
4. **What is the healing process of tendon to bone when it is repaired via suture anchors?**

## DISCUSSION

**Figure 1:**
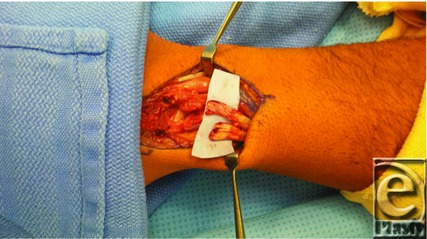
Avulsed extensor carpi radialis longus and brevis enthesis.

**Figure 2:**
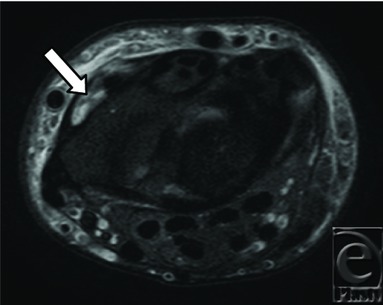
Axial T2 STIR, right wrist, demonstrating empty second dorsal extensor compartment (arrow).

**Figure 3:**
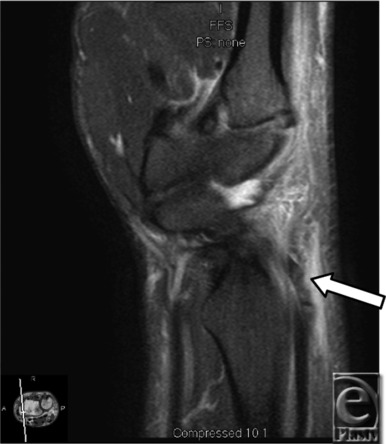
Sagittal T2, right wrist demonstrating avulsed second dorsal compartment (arrow).

**Figure 4:**
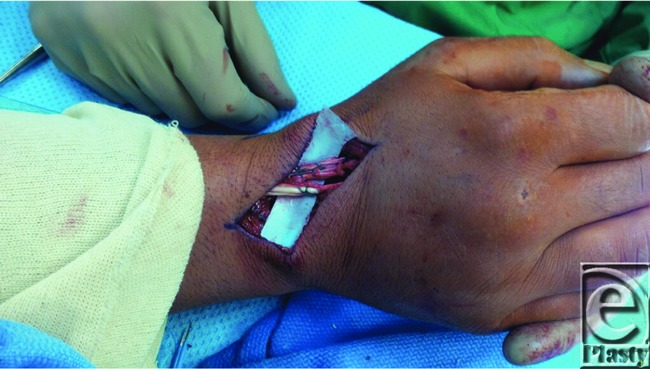
Intraoperative repair with mini-suture anchors.

There have been limited reports in the literature demonstrating avulsion injuries of both the ECRL and the ECRB tendons. Three cases reported traumatic avulsion of both ECRL and ECRB tendons^1^^,^^2^^,^^3^ after high-energy injury, and one case of isolated ECRB avulsion by similar mechanism as our patient.^4^ The most common mechanism of injury in acute tendon rupture is abrupt acceleration/deceleration^5^ secondary to their inherent viscoelastic properties. Our case demonstrates a traumatic avulsion of both the ECRL and ECRB (Fig 1) and repair via suture anchor (Fig 4). Magnetic resonance imaging reveals extensor pollicis longus crossing over an empty second dorsal compartment as well as evidence of avulsion from the base of the metacarpals (Figs 2 and 3).

Many studies describe the association of fluoroquinolone antibiotic use and tendon rupture, most commonly, the Achilles tendon. Patients taking fluoroquinolones, particularly ofloxacin, are at greatest risk of tendon rupture within the first month after taking the drug and are at increased risk of rupture if they are concomitantly taking a corticosteroid. Tendinitis is an independent risk factor associated with tendon rupture. There have been anecdotal reports of anabolic steroid use and creatine supplementation causing tendon rupture, although many questions remain. One report of weight-lifters showed no difference in collagen fibril structure under electron microscopy between those who used anabolic steroids and those who did not, while another study discovered a difference.^6^ Our patient has no documented history of fluoroquinolone, anabolic steroid, or supplement use. However, his body habitus was that of a body-builder.

Suture anchors were used to approximate the ECRL and ECRB tendons to the base of the second and third metacarpals, respectively. Suture anchors have various pullout strengths based on manufacturer and location, with mini suture anchors having pullout strength in metaphyseal porcine femur between 13 and 151 pounds of force.^7^ In general, suture anchors, especially when used in the hand, exceed pullout strength when compared to commonly used suture material by hand surgeons alone. Suture anchors provide more strength for tendon repair than suture material alone and are less likely to fail than other aspects of the repair. Suture anchors are strongest when inserted in the diaphysis (further from joint surfaces), when inserted in thick cortical bone, and when loading forces are applied parallel to the bone surface (ie, suture anchor inserted perpendicular to bone with loading force applied perpendicular to suture anchor).^7^

The tissue interface connecting tendon to bone is known as an enthesis. Entheses consist of 4 tissue zones (tendon, fibrocartilage, calcified fibrocartilage, and bone) that create a strong interface between bone and tendon. In our patient, the enthesis was likely interrupted as the ECRL and ECRB were avulsed from the bone surface with little visible tendon attachment remaining on the bone surface. To facilitate effective healing, the tendons must be approximated closely to their bony insertions, which can be accomplished using interference screws or suture anchors. The healing process is not clearly understood, but one study using sheep demonstrated formation of a fibrous bridge between the approximated tendon and bone, with histological differences from uninjured enthesis at 2 years.^8^

Our case demonstrates a rare injury, interesting imaging findings, and brings into question the most appropriate technique for surgical repair. Suture anchors demonstrate higher pullout strength when compared to suture alone; however, more studies are needed to understand exactly how the enthesis interface heals. The authors believe the treating physician should educate the patient on possible contributing factors to tendon rupture, which vary from pharmacotherapy to over the counter supplements.

## References

[1] Gurich RW, Tanksley JA, Pappas ND (2013). Late repair of combined extensor carpi radialis longus and brevis avulsion fractures. Orthopedics.

[2] Vandeputte G, De Smet L (1999). Avulsion of both extensor carpi radialis tendons: a case report. J Hand Surg Am.

[3] Boles SD, Durbin RA (1999). Simultaneous ipsilateral avulsion of the extensor carpi radialis longus and brevis tendon insertions: case report and review of the literature. J Hand Surg Am.

[4] Breeze SW, Ouellette T, Mays MM (2009). Isolated avulsion fracture of the extensor carpi radialis brevis insertion due to a boxer's injury. Orthopedics.

[5] Sharma P, Maffulli N (2006). Biology of tendon injury: healing, modeling and remodeling. J Musculoskelet Neuronal Interact.

[6] Tsitsilonis S, Panayiotis CE, Athanasios MS (2014). Anabolic androgenic steroids reverse the beneficial effect of exercise on tendon biomechanics: an experimental study. Foot Ankle Surg.

[7] Carpenter JE, Fish DN, Huston LJ, Goldstein SA (1993). Pull-out strength of five suture anchors. Arthroscopy.

[8] Newsham-West R, Nicholson H, Walton M, Milburn P (2007). Long-term morphology of a healing bone-tendon interface: a histological observation in the sheep model. J Anat.

